# Developing a Health Equity and Criminal Justice Concentration for a Master of Public Health (MPH) Program: Results From a Needs Assessment Among Community Partners and Potential Employers

**DOI:** 10.3389/fpubh.2019.00200

**Published:** 2019-07-25

**Authors:** Alexandra L. Hernandez, Mathew Green, Nemesia Kelly, Carly Strouse, Trina Mackie, Gayle Cummings, Elena O. Lingas

**Affiliations:** Public Health Program, College of Education and Health Sciences, Touro University California, Vallejo, CA, United States

**Keywords:** master in public health, MPH, criminal justice, curriculum concentration, mass incarceration, MPH concentration, health equity

## Abstract

The United States has experienced a 4-fold increase in jail and prison populations over the last 40 years, disproportionately burdening African American and Hispanic/Latinx communities. Mass incarceration threatens the health of individuals, families, and communities, and requires a public health response. The Master of Public Health (MPH) Program at Touro University California (TUC) trains students to become skillful, socially-conscious public health professionals. We are developing a concentration focused on the public health impacts of incarceration. Along with the core public health curriculum, students of this new “Health Equity and Criminal Justice (HECJ)” concentration will receive training in criminal justice, reentry, reintegration, recidivism, restorative justice, structural racism, and social and community impacts of incarceration. Our study gauges interest in an HECJ concentration in our local community, including potential employers. We surveyed a cross-section of community partners including public health departments, other governmental agencies, California correctional facilities, county jails, community groups, health clinics, and hospitals. A majority (89%) of respondents consider mass incarceration a public health problem and 86% believe specialized training would make graduates employable by criminal justice related organizations. The HECJ track will fill a gap in the field and train a future generation of public health professionals to address the epidemic of mass incarceration.

## Introduction

The number of incarcerated individuals in the United States (U.S.) has increased tremendously over the past 40 years with ~2.3 million individuals reported as incarcerated in 2016 (1), a level higher than any other high-income country. Incarceration disproportionately impacts African American individuals with African American men six times more likely to be incarcerated than non-Hispanic White men (2) and African American women twice as likely to be incarcerated as non-Hispanic white women (2). In 2016, Black and Hispanic/Latinx individuals represented only 28% of the adult population of the U.S. but accounted for 56% of incarcerated individuals, whereas Whites represented 64% of the adult population but only 30% of incarcerated individuals (3). These disparities extend into the non-incarcerated community. Forty-four percent of Black women report having a family member imprisoned compared to only 12% of White women (4). In a 2009 study, African American children born in 1990 had a 25% increased likelihood of having their father go to prison compared to non-Hispanic White children, and that figure rose to a 50% increase if their fathers had not finished high school (5). Although the disparities are not as striking as with African Americans, other persons of color are also overrepresented in U.S. jail and prison populations with the consequent impact on their families and communities (1, 2, 4–6).

This national picture is similarly reflected in California. In 2017, California prisons held over 115,000 individuals with African Americans representing 29% of the male prisoners, despite comprising only 6% of California's male population (7). The proportion of imprisoned African-American men in California is almost ten-times that of white men, and the population of imprisoned Latino men is almost twice that of white men (7). Despite similar rates of illicit drug use, African Americans are imprisoned at almost six times the rate of whites for similar infractions; and while African Americans represent only 12.5% of illicit drug users, they account for 29% of drug-related arrests (8). These data indicate the presence of institutional racism in the criminal justice system.

Incarcerated individuals of any race or ethnicity disproportionately suffer from a range of physical and mental health disorders: HIV is up to seven times more prevalent in prison populations compared to the general population; Hepatitis C is up to 21 times more prevalent (9). Mental health disorders are up to five times more prevalent in incarcerated populations than the general population, and ~68% of incarcerated individuals suffer from substance abuse disorders with only about 15% receiving adequate treatment (9). In addition, the population of incarcerated adults over 55 in the U.S. increased 550% from 1992 to 2012, prompting increased resources to attempt the management of chronic conditions and age-related disabilities (9).

The majority of those who are incarcerated are released, posing challenges for both reentry and making sure their health needs are addressed; for example in 2012 alone in California over 18,000 individuals were released on parole. With the 2014 passage of California Proposition 47 reducing sentences for certain crimes the number of persons re-entering their communities post-incarceration is projected to steadily grow. The California Department of Corrections and Rehabilitation (CDCR) estimated that in 2017 there were over 42,000 individuals in California on parole, 85% of whom will be eligible for Medi-Cal (10).

The economic and social impacts of incarceration on families and communities are also starkly apparent: incarceration severely diminishes the economic mobility of individuals, reducing their earnings by 40%, and thwarting potential for economic progress (6, 11). With 1 in 28 (or 2.7 million) U.S. children having an incarcerated parent, the lack of presence and economic support substantially impacts child development (11). Moreover, individuals living in neighborhoods with higher rates of incarcerated individuals face increased risks for major depressive disorder and generalized anxiety (12).

Incarceration negatively impacts the physical, mental, emotional, and financial health of the individuals incarcerated, and families and communities impacted by incarceration and its sequelae (6). African Americans are disproportionately affected by incarceration, impacting racial disparities in health outcomes. For all of the above reasons, mass incarceration is a significant problem for public health. Notably, to our knowledge no Council on Education for Public Health (CEPH) accredited Master of Public Health (MPH) program offers a concentration on the public health impacts of incarceration.

The graduate Public Health Program at Touro University California (TUC) educates and empowers diverse cohorts of students to become skillful, socially-conscious public health professionals through a robust course of academic and professional training. Our students consist of traditional Master of Public Health (MPH) students as well as students obtaining clinical degrees in Osteopathic Medicine (DO), Pharmacy (PharmD), and Physician Assistant studies (MSPAS). In addition to covering the five core disciplines of public health—Biostatistics, Epidemiology, Environmental Health Science, Health Policy and Management, and Social and Behavioral Sciences, the program's 42-unit curriculum also consists of a Public Health Field Study (public health practicum), a culminating Capstone Project, Breadth courses, and 10 units of coursework in either the Community Action for Health (formerly Community Health) or Global Health concentrations. The concentrations differ in two didactic courses and the location of the field site. Students choose which concentration to follow based on their professional interests, and are prepared for public health careers in a variety of local and global settings.

In 2017 the TUC MPH program began to explore the possibility of adding an additional MPH concentration to address the gap in training on the health impacts of incarceration on individuals, families, and communities. Students choosing this new concentration, entitled “Health Equity and Criminal Justice (HECJ),” would receive special training in criminal justice, reentry, recidivism, and social and community impacts of incarceration along with the core Public Health curriculum. The concentration would also include a field study placement at a California correctional facility or community organization working with individuals, families, and communities impacted by incarceration.

As a preliminary step in developing this concentration, we surveyed our community partners and potential employers of MPH graduates in and around our university to gain further information on the utility of the program, career potential, current gaps in student knowledge, skills and training needed, and general interest in this new concentration. This study presents the results of that survey.

## Methods

We conducted an anonymous cross-sectional survey designed for members of our community who could potentially employ or otherwise work with students who graduate with an MPH with a concentration in HECJ. Our target population consisted of current partners of the TUC Public Health Program (TUC-PHP) who hosted students during their field studies and those interested in becoming partners. This included individuals at city and county health departments, other government agencies, California correctional facilities, county jails, community groups, health clinics or hospitals, or other non-profit organizations. The survey was sent out to all of our existing field placement partners as well as our new incarceration-related contacts (150 individuals). Separately, we also compiled a list of California non-profit groups who were working with individuals impacted by incarceration through Internet searches, phone calls, and word of mouth (~30 groups). The survey also asked respondents to recommend another person who might be interested in completing the survey, and we forwarded the survey link to all of the recommended individuals. We also posted the survey link to our TUC-PHP Facebook page and asked our community partners to respond.

### Survey

Our survey was developed in Qualtrics and consisted of yes/no, multiple choice, Likert scale, and open-ended questions. We included a few demographic factors to describe our population. The majority of the questions were designed to gauge gaps in training, knowledge, and skills among individuals with MPH degrees to address the unique challenges faced by incarcerated and formerly incarcerated individuals, families, and communities. The questions were developed based on literature review and dialogue with content experts. We also asked about the utility and potential career opportunities available to those with MPH degrees with a concentration in Health Equity and Criminal Justice, and to identify key topics to include in our curriculum.

The survey was distributed to the email lists and on Facebook from August 2017 to September 2017.We offered a chance to receive a $50 Amazon.com gift card for participation in the survey. A copy of the survey is included as **Supplement A**.

### Analysis

All quantitative variables were summarized with frequency counts and proportions. The open-ended fields were analyzed as qualitative data by parsing out themes.

We asked our respondents their opinion on a number of statements about the U.S. criminal justice system and public health on a five point Likert scale with options of Strongly Disagree (SD) to Strongly Agree (SA).

We asked respondents to describe their gender in an open-ended question. If respondents listed the exact word “Male,” “Man,” or “Boy” we included them in the “Male” category. If respondents listed the exact text as “Female,” “Woman,” “Girl,” “cis Woman,” or “F” we included them as “Female.” No one self-reported a different gender identity.

We also asked our participants to describe their race and/or ethnicity in an open-ended question. If respondents listed “Asian,” “Asian, Korean,” “Vietnamese,” “East Indian” we included them in the “Asian” category; “African American” or “Black” we included them in the “African American” category; “Multiracial,” “Mixed race,” “Mixed, Asian,” “biracial,” “Mixed—White and Native American,” or “Asian/White” we included them in the “Multiracial” category; “White/European American,” “White,” “Caucasian” we included as “White”; “Latina,” “Hispanic” we included as Latino/Hispanic and “Other” we included as “Other.”

All methods were approved by the Touro University California Institutional Review Board (IRB) before initiation of the project.

## Results

We received 36 anonymous responses to our survey. Thirty-three percent of respondents reported their gender as “male,” and 67% “as female” (Table 1). Forty-seven percent of respondents reported “White,” 19% “African-American,” 8% “Asian,” 17% “Multiracial,” 6% “Latino,” and 3% “Other” in an open-ended question “please describe your race or ethnicity.” Forty-four percent of respondents were over 45 years of age, and 72% had completed graduate school. Two (6%) of the responding organizations were directly involved in the criminal justice system (CA Correctional Facility and Law Enforcement), but all respondents indicated some interest or involvement with either individuals, families, or communities impacted by incarceration.

**Table 1:** Participant characteristics.

**Characteristic**	**N**	**(%)**
**Total**	**36**	
Gender^*^		
Male	12	33%
Female	24	67%
Race/ethnicity^**^		
White	17	47%
African American	7	19%
Multiracial	6	17%
Latino	2	6%
Asian	3	8%
Other	1	3%
**Age**		
<25	1	3%
25-35	8	22%
36-45	10	28%
45+	16	44%
Decline	1	3%
**Education**		
Less than college	1	3%
Completed undergraduate	9	25%
Completed Graduate	26	72%
**What type of organization do you work for?**		
Health Care (hospital/clinic)	8	22%
Community Based Organization	6	17%
State Agency	2	6%
Regional/Local Government	2	6%
CA Correctional Facility	1	3%
Law Enforcement	1	3%
Local Health Department	8	22%
Other	8	22%
**Hiring manager**		
Yes	13	36%
No	23	64%

* *Participants were asked “Please describe gender” in an open field*.

** *Participants were asked “Please describe your race and/or ethnicity” in an open field*.

For all statements about the U.S. criminal justice system and public health (Table 2), at least 70% of respondents indicated that they “Strongly Agreed (SA)” with the statement. The statement with the highest proportion of respondents indicating SA was “Mass incarceration is a public health problem” (89%). Eighty-six percent of respondents selected SA for the statement “Race/ethnicity impacts who gets arrested, prosecuted and incarcerated” and 86% said SA for “Health equity and criminal justice are an integral part of public health.” The statement with the lowest proportion of SA was “Families of incarcerated and previously-incarcerated individuals are more likely to experience mental health issues such as depression,” however the proportion that selected SA was still high at 72%.

**Table 2:** Opinions regarding Public Health and Incarceration.

	**Strongly disagree (%)**	**Disagree (%)**	**Neutral (%)**	**Agree (%)**	**Strongly agree (%)**
Mass Incarceration is Public Health problem	0%	3%	3%	6%	89%
Race/Ethnicity impacts who gets arrested, prosecuted, and incarcerated	0%	0%	3%	11%	86%
Economic Status/class impacts who gets arrested, prosecuted, and incarcerated	0%	0%	3%	17%	81%
Previously-incarcerated individuals have poorer health outcomes than the general population in their community	0%	0%	3%	19%	78%
Immigration status impacts who gets arrested, prosecuted, and incarcerated	0%	3%	14%	8%	75%
Educational status impacts who gets arrested, prosecuted, and incarcerated	0%	0%	8%	14%	78%
Families of incarcerated and previously-incarcerated individuals are more likely to experience mental health issues such as depression	0%	0%	0%	28%	72%
Health equity and criminal justice are an integral part of Public Health	3%	3%	0%	8%	86%

*Participants were asked to rate their level of agreement to the following statements (regarding the United States)*.

We asked respondents to select which topics would be important to include in the curriculum for MPH students with a concentration in HECJ (Table 3). The majority of respondents indicated that all of the topics would be important to include. “Incarceration impacts on families” had the most positive responses at 94%, followed by “Incarceration impacts on communities” (89%), and “chronic health impacts of incarceration” (89%). None of the topics received <64% positive response. Respondents were also asked to recommend other topics that were not included on our list of choices. We compiled themes from the responses (Table 4). Many respondents listed institutional racism, mental health, policy and advocacy, social determinants of incarceration, and substance abuse as important topics to consider.

**Table 3:** Topics, skills, and employability of students earning an MPH specializing in HECJ.

**Characteristic**	**N**	**(%)**
**What topics do you believe are especially important to cover in training students in Health Equity and Criminal Justice? (multiple responses possible)**		
Incarceration impacts on families	34	94%
Incarceration impacts on communities	32	89%
Chronic health impacts of incarceration	32	89%
Economic impacts of incarceration	31	86%
Health of incarcerated individuals	31	86%
Historical contributors	31	86%
School to prison pipeline	28	78%
Recidivism	28	78%
Environmental contributors	27	75%
The U.S. criminal justice system	26	72%
Criminology (who commits crimes and why)	25	69%
Detention systems for immigrants-past and current	24	67%
Penalization and crime classification	23	64%
Other (Included in Table 4)	16	44%
**Do you think training Master of Public Health (MPH) students in Health Equity and Criminal Justice would make them employable by organizations such as yours?**		
Yes	31	86%
No	5	14%
**How employable do you think an MPH graduate with an emphasis in Health Equity and Criminal Justice would be compared to an MPH graduate with an emphasis in Community Health?**		
Less Employable	5	13.9%
Equally Employable	12	33.3%
Somewhat More Employable	7	19.4%
Much More Employable	8	22.2%
Decline	4	11.1%
**How employable do you think an MPH graduate with an emphasis in Health Equity and Criminal Justice would be compared to an MPH graduate with an emphasis in Global Health?**		
Less Employable	3	8.3%
Equally Employable	15	41.7%
Somewhat More Employable	7	19.4%
Much More Employable	9	25.0%
Decline	2	5.6%
**Could any of your employees benefit from an MPH with an emphasis in Health Equity and Criminal Justice?**		
Yes	26	72.2%
No	6	16.7%
Decline	4	11.1%

**Table 4:** Additional thematic areas generated from Community Partners and Preceptor survey responses suggested by at least one survey participant as being important to include in training for students in HECJ Concentration.

**Theme**	Number of times mentioned^*^
Institutional racism and historical context of incarceration	9
Mental health	8
Basics of criminal justice system	8
Policy and advocacy	8
Statistics/trends of disparities/social determinants incarceration	7
Substance abuse	5
Prison health system and health of prisoners	5
Impact to family members	5
Racial/cultural humility, self-awareness of own background	4
Post incarceration and reintegration	4
Culture/values of sheriffs, deputies, guards etc.	3
Innovative intervention models in and outside of prison/jail	3
Women and children's health/pregnancy	2
Prison-Industrial economics	1
Restorative justice^**^	1
Implicit bias^**^	1

* *Respondent may have mentioned multiple topics and can be represented in multiple themes*.

** From question “What additional education/training would they benefit from?”

Survey respondents were asked if they felt that the HECJ concentration would make MPH graduates more or less employable than a Community Health concentration or a Global Health concentration (Table 3). For each question about half said that students would be “somewhat” or “more” employable than the other traditional MPH concentrations. Fifteen percent of respondents believed that MPH graduates with a concentration of HECJ would be less employable than a Community Health concentration and 9% said they would be less employable than the Global Health concentration. When asked why they thought students would be less employable, several respondents indicated that the concentration would be “too narrow” or “too specialized.” For example, “Unless an organization is working with the incarcerated population the HECJ graduate may appear to be too narrow of a focus.” Those answering in comparison to Global Health indicated that the HECJ track would not translate to international settings given the large differences between the U.S. and other nations' criminal justice systems.

Respondents were asked to volunteer what skills MPH graduates should have with a concentration in HECJ. Of those who responded, almost all indicated that students should get experience working in jails, prisons, courts, with the formally incarcerated, or families/communities of the incarcerated, followed by data analysis skills. Several respondents also listed training in conducting focus groups, advocacy, and community organizing (Table 5).

**Table 5:** Skills and job opportunities identified by community partners and preceptors.

**Additional skills suggested by survey participants that would be important to include in training for students in HECJ concentration**.	Number of times mentioned^*^
Experience working in jails, prisons, courts, or with those formally incarcerated	20
Data collection and analysis	6
Conducting focus groups and interviews	4
Advocacy and community organizing	3
**What types of job opportunities would be available within your organization for someone with an MPH in HECJ concentration?**	Number of times mentioned^*^
Director/coordinator/administrator/project manager	9
Health educators/outreach/community health worker/health navigator	6
Case manager/mental Health	6
Data analyst/research associate/research scientist	5
Policy advocate, grant writer	3
Health care provider	3
Quality improvement analyst, evaluator	2

* *Respondent may have mentioned multiple topics and can be represented in multiple themes*.

When asked what type of education and training current employees of the responding organizations had specific to corrections or criminal justice, most responded that they had no specialized training, several responded “experience” or “on the job training” and several listed degrees (“masters,” “law degrees,” “clinicians”). Seventy-two percent reported that current employees could benefit from an MPH with a concentration in Health Equity and Criminal Justice, indicating that the employers see value and necessity of such training for a competent workforce.

When asked what types of job opportunities would be available within the respondent's organization, respondents volunteered: Directors/coordinator/administrators/ or managers of programs, data analysts/research associates or research scientists, and health educators, outreach, community health workers, or health navigators (Table 5).

## Discussion

Data collected in our cross-sectional survey provide evidence to support the development and implementation of a public health curriculum focused on health equity and criminal justice. As current research indicates, the complicated, and multi- dimensional issues surrounding criminal justice and mass incarceration are directly related to social determinants of health, such as racism, poverty, and inadequate education (13). Given that public health as a discipline recognizes social determinants of health as one of the primary causes of health inequalities between different population groups, there appears to be a clear interplay between existing public health training and criminal justice prevention, policies and practices.

A major survey finding is that 89% of respondents believe mass incarceration to be a public health problem. This finding supports a widening consensus in emerging literature describing mass incarceration as a public health problem (6, 13–15). Research in this area focuses on the mental and physical health impacts of incarceration on citizens who regularly interact with the criminal justice system (14) as well as on the broader community impacts of mass incarceration for families and children (6).

Eight-six percent of respondents believe MPH training in health equity and criminal justice would make graduates employable by criminal justice related organizations. This finding is supported by the current workload of public health departments in California. A study investigating California's County Public Health Departments' current engagement in criminal justice related issues found that health departments in every county throughout the state are currently involved in some level of the criminal justice system (13). These services includes direct delivery of health services, re-entry services and programs, diversion programming, prevention services in schools, primary prevention services, and access to health insurance for released citizens (13). With the implementation of this new HECJ concentration, TUC MPH graduates will be primed to strengthen and expand the current efforts underway in county public health departments to address the public health impacts of mass incarceration.

We believe the skills gained in this new concentration will be transferable to public health work in many communities in the U.S. because incarceration touches the lives of almost every American in one way or another. This concentration is bringing the context of the health impacts of incarceration into clearer focus. Our study findings support the idea that graduates with this special concentration will also be able to fill “traditional' public health positions within organizations. Positions included, directors, research associates, data analysts, health educators, case managers, and policy advocates.

The small sample size and convenience sampling are the primary limitations of this study. However, this was an exploratory study to gauge employer interest in the new concentration and these data will be used to inform our curriculum development. We surveyed a convenience sample of organizations that had connections to our public health program and may already share our opinions on the importance of health equity and criminal justice in our community. Furthermore, as we were interested in organizations that could potentially employ our graduates, the employers who responded to our survey would be some of the first contacts that our graduates would seek out in their job search.

Our findings clearly suggest that MPH graduates with specialized training in HECJ would be employable across both traditional public health and criminal justice sectors. We feel confident that our program is filling a gap in our field and training a future generation of public health professionals to serve this marginalized population.

## Data Availability

The raw data supporting the conclusions of this manuscript will be made available by the authors, without undue reservation, to any qualified researcher.

## Ethics Statement

This study was carried out in accordance with the recommendations of the Touro University California Institutional Review Board with electronic informed consent from all subjects on the first page of the survey. All subjects gave electronic informed consent in accordance with the Declaration of Helsinki. The protocol was approved by the Touro University California Institutional Review Board.

## Author Contributions

AH designed project, surveys, analyzed and interpreted data, and drafted manuscript. MG contributed to survey design and implementation, analyzed and interpreted data, and drafted manuscript. NK, CS, TM, and GC contributed to project design and reviewed and edited manuscript. EL contributed to the design of project and survey, interpreted data, reviewed, and edited the paper.

### Conflict of Interest Statement

The authors declare that the research was conducted in the absence of any commercial or financial relationships that could be construed as a potential conflict of interest.
